# Development of Hausa dataset a baseline for speech recognition

**DOI:** 10.1016/j.dib.2022.107820

**Published:** 2022-01-10

**Authors:** Umar Adam Ibrahim, Moussa Mahamat Boukar, Muhammed Aliyu Suleiman

**Affiliations:** Faculty of Natural and Applied Sciences, Computer Science Department, Nile University of Nigeria, Abuja, Nigeria

**Keywords:** Corpus, Automatic speech, NLP, Text-to-speech, Hausa corpus

## Abstract

The Hausa language read-speech dataset was created by recording native Hausa speakers. The recording took place at Nile university of Nigeria audio studio and radio broadcasting studio. The recorded dataset was segmented into unigram and bigram. The Hausa speech dataset contain 47hr of recorded audio speech. The dataset can be used for automatic speech recognition, speech synthesis, Text-to-Speech and speech-to-text application.

## Specifications Table

**Table:** 

Subject	Computer Science, Human-Computer Interaction, Computer Science Applications
Specific subject area	Human-Computer Interaction is the ability for user to interact with the computer. This include the ability of human to talk to his/her phone, computer and other smart devices. In this area of human talking to their devices there is need for a readily available dataset that can be used to create applications and system that enable human to interact with their devices.
Type of data	Speech
How the data were acquired	*The datasets were acquired by recording volunteers via microphone. Some recording took place at Nile University of Nigeria Audio Studio, some at Radio Broadcasting Audio Studio and other in quiet room at homes via phone microphone.* *The following tools was used:* *Microphone* *Headset* *Audio Console* *HP Computer* *Load Speaker Monitors* *Adobe Edition Software*
Data format	Raw Filtered segmented Labelled
Description of data collection	While selecting the literatures the criteria adopted are grammatical richness, culture representation, terminology and the genre of the book. Nine Hausa literature books was selected. Some of these books were recorded at Nile University Audio Studio, while some at Radio Broadcasting studio and some at in quiet rooms at Home. The recorded raw data was later segmented into unigram (one word) and bigram (two word).
Data source location	• Institution: Nile university of Nigeria • City/Town/Region: Jabi Abuja • Country: Nigeria
Data accessibility	Repository name: Mendeley Data Data identification number: 10.17632/z3f8hsttxb.1 Direct URL to data: https://data.mendeley.com/datasets/z3f8hsttxb/1

## Value of the Data

• The data presented is important because it is the first freely licensed Hausa speech dataset.
• The dataset is useful because it can used in developing Automatic Speech recognition, Text-to-Speech system, Speech-to-Text for Hausa Language.
• Researcher can use the dataset for speech research for Hausa language. While the industry can use the data in developing applications for Hausa language.
• The present dataset is in raw state that can be further segmented into trigram as we have segmented the data in unigram and bigram.

## 1 Data Description

Almost every language has writing system. Hausa has two writing system Ajami (originated from Arabic scripts) and Boko (Originated from Latin scripts). Hausa is a Chadic language family that is within the family of Afro-Asiatic languages [1]. The dataset uploaded in the repository contain nine different literature recordings. The literatures were recording by different people at different locations. These locations are Nile University Audio Studio, Radio Broadcasting studio and Quiet Room at different homes.

The aim of constructing HSC is to provide a public Hausa language corpus that will serve as a baseline corpus for ASR research and application [2]. More also to promote research in Hausa speech processing applications. Although some Hausa speech corpora were collected and developed in [3] and [4], they are publicly not available. Besides, they are insufficient to train and build a reliable end-to-end model. These collected corpora are publicly unavailable or contain an insufficient amount of data to train reliable models, which are extremely data hungry [5].

The dataset uploaded in the repository contain nine different literature recordings. The literatures were recording by different people at different locations. These locations are Nile University Audio Studio, Radio Broadcasting studio and Quiet Room at different homes.

At Nile University, audio studio the book Iliya dan Maikarfi and Koya da kanka was read. 154 recordings took place at the Nile university studio. At broadcasting studio, the following literatures were read: Shehu Umar, A Duniya Ne, Rayuwar Hibba, Komai Nisan Dare, Gani Gare Ka, Wani Gari Yafi Gaban Kunu, Magana Jarice, Jiki Magayi. 167 recordings took place at broadcasting. While Kamus Na Turanci Da Hausa was read in, quit rooms by different people. 36 recordings was done at home. The raw dataset are presented in dot mp3 format. The place of recording, recording time and the gender of recorder for each literature was presented in Table 1. The literatures were chosen because they contain rich Hausa vocabulary and grammar. For the Kamus Na Turanci da Hausa it is a Hausa-English dictionary which recordings was done for machine translation project. While Koya Da Kanka is a mini Hausa book the contain Hausa daily conversation and digits.

**Fig. 1:**
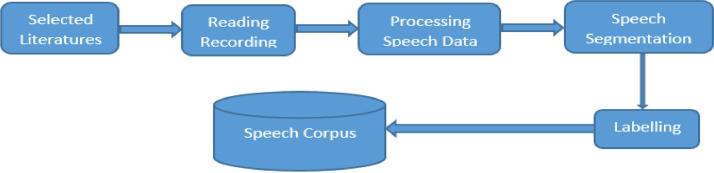
Speech corpus development flow.

Fig. 1. Depict the speech corpus development flow.

**Fig. 2:**
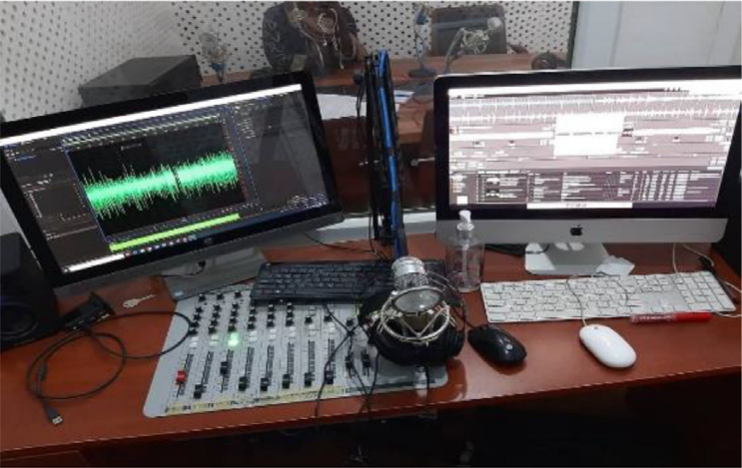
Nile University studio setup.

Fig. 2. Presented the recording device used at the studio

**Fig. 3:**
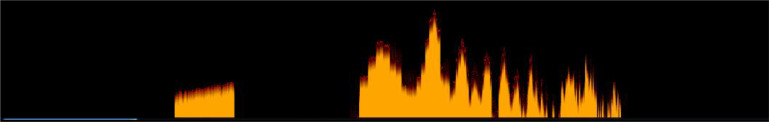
Sentences-words-syllables of Hausa.

Fig. 3. Shows the waveform of a selected sentence-word syllables of Hausa.

**Fig. 4:**
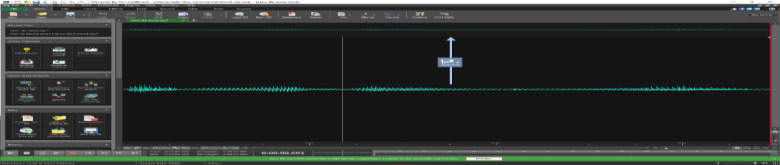
Sample audio of a single sentence.

Fig. 4. Presented the a sample of audio of a single Hausa sentence.

Table 1. Represent the selected literature, where the book name, genre, place of recording and naming conversion was presented.

**Table 1:** Selected literatures.

s/n	Name	Genre	Place of Recording	Naming Conversion
*1*	*Koya Da Kanka*	*Daily Conversation*	*Nile Studio*	*ns_kdk_f_001*
*2*	*Ilya Dan Maikarfi*	*Fiction*	*Nile Studio*	*ns_idm_f_001*
*3*	*Kamus Na Turanci Da Hausa*	*Dictionary*	*Quiet Rooms*	*qr_kth_f_001* *qr_kth_m_001*
*4*	*Shehu Umar*	*Biographies History*	*Broadcasting studio*	*bs_su_f_001*
*5*	*A Duniya Ne*	*Fiction*	*Broadcasting studio*	*bs_adn_f_001*
*6*	*Rayuwar Hibba*	*Texts*	*Broadcasting studio*	*bs_rh_f_001*
*7*	*Komai Nisan Dare*	*General fiction*	*Broadcasting studio*	*bs_knd_f_001*
*8*	*Gani Gare Ka*	*Fiction*	*Broadcasting studio*	*bs_ggk_f_001*
*9*	*Wani Gari Yafi Gaban Kunu*	*Drama*	*Broadcasting studio*	*bs_wgygk_f_001*
*10*	*Magana Jarice*	*Lore*	*Broadcasting studio*	*bs_mj_m_001*
*11*	*Jiki Magayi*	*Fiction*	*Broadcasting studio*	*bs_jm_f_001*

Table 2. Presented the recorded time for each literature in hours and minutes.

**Table 2:** Recordings and locations.

s/n	Location	Recordings
*1*	*Quiet Rooms at Home*	*36*
*2*	*Audio studio - Nile University of Nigeria*	*154*
*3*	*News Broadcasting studio - Kaduna*	*167*
*4*	*Total*	*357*

Table 3. Presented the recordings and locations.

**Table 3:** Recorded time.

s/n	Literature	Hours	Minutes
*1*	*Koya Da Kanka*	*0*	*11*
*2*	*Ilya Dan Maikarfi*	*0*	*32*
*3*	*Kamus Na Turanci Da Hausa*	*5*	*20*
*4*	*Shehu Umar*	*2*	*21*
*5*	*A Duniya Ne*	*11*	*29*
*6*	*Rayuwar Hibba*	*8*	*15*
*7*	*Komai Nisan Dare*	*2*	*5*
*8*	*Gani Gare Ka*	*10*	*13*
*9*	*Wani Gari Yafi Gaban Kunu*	*3*	*9*
*10*	*Magana Jarice*	*2*	*32*
*11*	*Jiki Magayi*	*1*	*30*

Table 4. Presented the statistical summary of the n-grams after segmentation.

**Table 4:** Statistical summary of the *n*-grams after segmentation.

s/n	Gram Type	Number of files
1	Unigram(one-word)	34,000
2	Bigram(two-words)	16,000

## 2 Experimental Design, Materials and Methods

Gutkin et al. [5] developed an open-source Yoruba speech corpus. Yoruba is a language spoken in western African. It is part of Niger-Congo family. The authors recorded 36 male and female using two offices in Lagos Nigeria. The corpus was used for Text-to-Speech application by implementing Hidden Markov Model.

Georgescu et al [6] developed the largest Romanian speech corpus. The came up with 100hours speech corpus read by 164 people. It is one of the large public data set for Romanian speech corpus. They collected their corpus from interview, news and literature utterances. They presented the acoustic and language model of the Romanian speech corpus. For model analysis the used RNN language based.

The authors took advantage of the Catalan TV program. This program also have transcribed files attached to them. Their aim of using public TV program was to reduce the cost of collecting and transcribing the data set [7].

Selecting the accurate length of script from an audio set is an important feature in Text-to-Speech application as presented by [8].

Paper [9] describes the experiences and challenges while collecting speech data for Hindi via mobile phone. The goals in [10] was to enhance speech data transcription and harvesting accuracy, enhance text normalization process and pronunciation modelling. According to [11] the major challenges when it comes to speech corpus creation is segmenting audio data into sentences. [12] presented the methodology the used for designing and creating Hindi speech corpus. The methodology involved crawling text, filtering, recording and annotation phases.

The Hausa speech corpus dataset collected was recorded in three different locations viz., quiet room at homes, university audio studio and news broadcasting studio. The dataset comprised of 357 files in mp3 format. Females and Males readers participated voluntarily. The readers were informed of the dataset collection and use protocols.

### 2.1 Study area

The literatures were selected based on the generic and specific nature of the literature. Volunteers were contacted for recording purposes. The recording took place in three different locations.

### 2.2 Data acquisition

Some of the recording took places at Nile University of Nigeria Mass Communication Department Audio Studio. Other recording to place at broadcasting studio whilst some of the recording took place in quiet room at different homes. Just as paper [15] presented the process of developing speech corporal, pronunciation dictionary and transcription for Malayam language automatic speech recognition. The research started by building a text corpus. The corpus contained connected and isolated digits. These would be used for recognition task. Additionally, the corpus contained text data for continuous speech recognition task. The data was recorded in an office environment via microphone. The 15 speakers were asked to read in a normal reading manner. The data set contain Malayam unique phoneme categories and phoneme classes, which was used for analysis.

### 2.3 Data processing

Paper [13] described the system architecture for the development of speech corpus. The paper also described acoustic-phonetic approach of developing speech corpus. Work done in [14] presented a method that can automatically structure text files of Filipino audio files for deep learning automatic speech recognition.

The adobe Audition software was used for recording. For pre-processing spectral subtraction and adaptive noise cancellation algorithm was applied [18]. The aim is to remove background or ambient noise. This is adjust or modify speech signal and the create feature vectors. Some of the recordings were filter out due to errors in pronunciation, noise in background and lack of clarity in the readers’ voices. The dataset composed 11 different folders with files in mp3 format. The dataset has 47 hr of spoken Hausa sentences, words and syllables with a file size of 3.97 GB. Each file in the folder follows a naming convention used to distinguished one file from another.

The raw dataset needs to pre-processing to make it ready for speech recognition. To manipulate this waveform signal, the data needs to be translated into spectrograms. Waveform is a visual representation of spectrum of frequencies of a signal as it varies with time. Adobe audition was used as an editing tool to do simple cut and splicing needed for this speech manipulation.

A sample sentence waveform signal was taken from one audio file and spliced using wavepad. The transcript being uttered in the Hausa sentence is “Sannu Da Zuwa” (English translation: “Welcome”) [17] work, which stated that “audio segmentation for achieving accurate alignment”. The audio segmentation target was to improve baseline acoustic model by improving the quality of text normalization and the accuracy of the pronunciation dictionaries. To perform the speech segmentation, Adobe audition was installed. The recorded speeches were segmented into sub-words and words. Segmentation is just the act of breaking down a continuous speech into discrete units like words, phonemes, syllables and meaningful sub-words [16]. These segmented speech utterances can be used while building automatic speech recognition system.

## Ethics Statements

The readers were contacted, informed of the dataset collection process and use protocol. Permission was also seek from the head of department, Mass communication Nile university of Nigeria.

Permission was also seek for recording done in broadcasting studios.

## CRediT authorship contribution statement

**Umar Adam Ibrahim:** Conceptualization, Methodology, Data curation, Writing – original draft. **Moussa Mahamat Boukar:** Supervision. **Muhammed Aliyu Suleiman:** Writing – review & editing.

## Declaration of Competing Interest

The authors declare that they have no known competing financial interests or personal relationships that could have appeared to influence the work reported in this paper.
